# Contribution of Energy Dysfunction to Impaired Protein Translation in Neurodegenerative Diseases

**DOI:** 10.3389/fncel.2021.668500

**Published:** 2021-07-28

**Authors:** Yu-Ju Liu, Yijuang Chern

**Affiliations:** Division of Neuroscience, Institute of Biomedical Sciences, Academia Sinica, Taipei, Taiwan

**Keywords:** AMP kinase, protein translation, Alzheimer’s disease, amyotrophic lateral sclerosis, stress granules

## Abstract

Impaired energy homeostasis and aberrant translational control have independently been implicated in the pathogenesis of neurodegenerative diseases. AMP kinase (AMPK), regulated by the ratio of cellular AMP and ATP, is a major gatekeeper for cellular energy homeostasis. Abnormal regulation of AMPK has been reported in several neurodegenerative diseases, including Alzheimer’s disease (AD) and amyotrophic lateral sclerosis (ALS). Most importantly, AMPK activation is known to suppress the translational machinery by inhibiting the mechanistic target of rapamycin complex 1 (mTORC1), activating translational regulators, and phosphorylating nuclear transporter factors. In this review, we describe recent findings on the emerging role of protein translation impairment caused by energy dysregulation in neurodegenerative diseases.

## Introduction

Despite the tremendous effort that has been devoted to the investigation of neurodegenerative diseases in the past decades, effective treatments remain limited. Among the common features of most degenerative diseases, impaired energy homeostasis due to mitochondrial defects and reduced protein translation efficiency are commonly reported. Ample evidence has shown that boosting bioenergetics in neurons may provide beneficial effects in various experimental models of neurodegenerative diseases, suggesting the importance of energy deficiency in diseased brains. Because the AMP-activated protein kinase (AMPK) is a key sensor of cellular energy status and an upstream regulator of multiple cellular machineries, its functions during energy stresses have been well investigated. Conversely, the protein translation process is highly energy- dependent and tightly regulated by hundreds of proteins at multiple steps under various pathophysiological conditions. In particular, gradual loss of protein translation efficiency along the progression of neurodegenerative diseases has been reported. This mini- review summarizes the current understanding of the role of energetic stress-mediated translational dysfunction in neurodegenerative diseases. Our focus is on the molecules and/or pathways in protein translation that are disturbed by energy deficiency and aberrant AMPK activation in neurodegenerative diseases, with a specific focus on Alzheimer’s disease (AD) and amyotrophic lateral sclerosis (ALS).

## Protein Translation Is Energy- Dependent and Tightly Regulated at Multiple Steps

**Figure 1:**
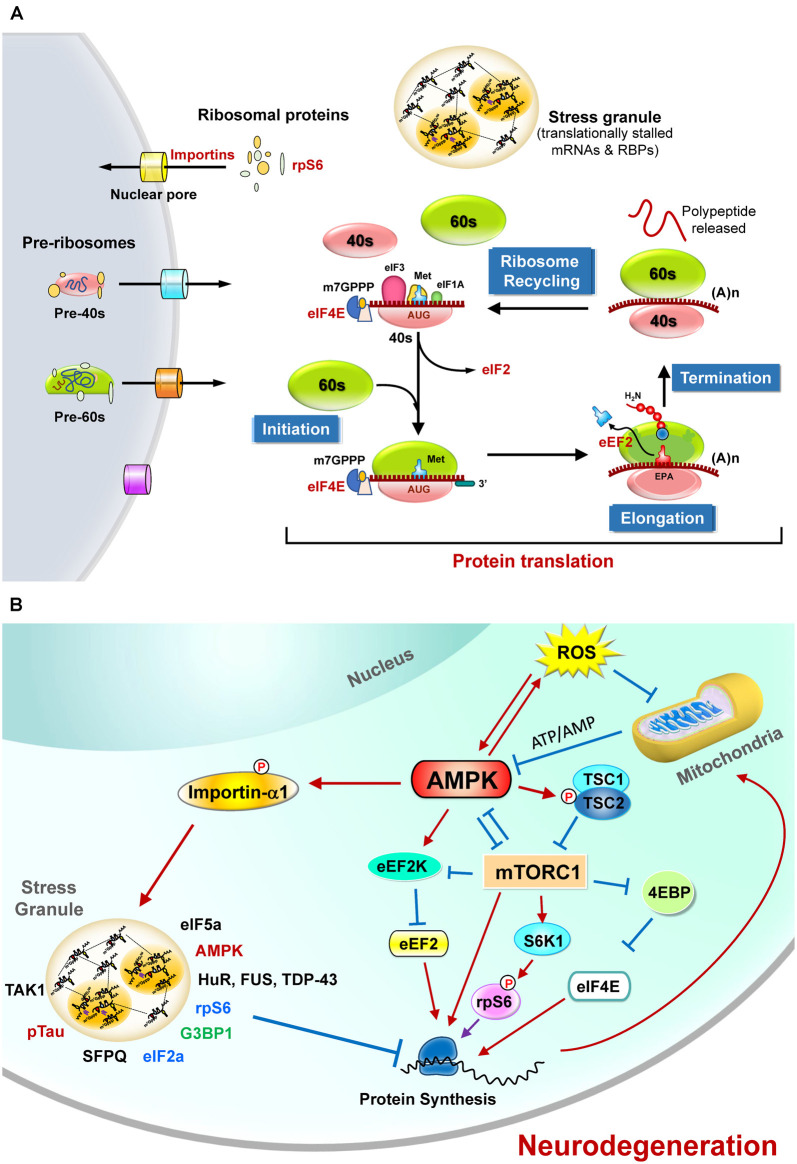
The regulation of protein translation by energy deficiency and AMPK activation at multiple steps.** (A)** Schematic representation of the four critical steps to control eukaryotic protein translation. For the sake of clarity, only those sensitive to energy homeostasis are listed here. The initiation stage involves the start codon recognition and the joining of the ribosomal subunits (40S and 60S), followed by the elongation stage with repetitive binding of aa-tRNA and translocation. When the ribosome complex encounters stop codons (UAA, UGA, or UAG), protein synthesis is terminated with the help of a protein release factor at the termination stage. The ribosome complex is then dissociated into ribosomal subunits in the ribosome recycling stage and transported into nuclei by importins. These steps are controlled by hundreds of proteins including various initiation factors (eIF) and elongation factors (eEF). In response to stress stimuli that reduce protein translation efficiency, the formation of stress granules (SGs) containing translationally stalled mRNAs and RNA-binding proteins (RBPs) can be observed. **(B)** The protein translation process is regulated by mitochondrial defect and AMPK activation. Briefly, elevated reactive oxygen species (ROS) and the mitochondrial defect may cause AMPK activation and subsequently inhibit protein translation by suppressing the functions of eIF4E, rpS6, s and eEF2. Activation of AMPK also causes phosphorylation of importin-α1 and interrupts its function in mediating nucleocytoplasmic transport of several RBPs (including TDP-43, HuR). During these stresses, importin-α1 can be found in SGs and is believed to play an important role in the assembly of SGs for the temporary repression of protein translation. A wide variety of proteins (including the components of translation apparatus, RBPs, ribosomal proteins, and pTau) are also recruited to SGs under stresses that cause neurodegeneration. See text for additional details.

Eukaryotic protein translation is tightly controlled by hundreds of proteins at four steps (i.e., initiation, elongation, termination, and ribosome recycling (Hershey et al., 2019; Figure 1A). For the sake of clarity, only those sensitive to energy homeostasis are elaborated in the following sections. The main protein translation apparatus in mammalian cells is the 80S ribosome, which is composed of 40S and 60S ribosomal subunits. These ribosomal subunits contain four ribosomal RNA species and 79 ribosomal proteins (Kressler et al., 2010). The assembly of ribosomes occurs mainly in the nucleus, with some exceptions in the axon (Bohnsack and Bohnsack, 2019; Shigeoka et al., 2019). Controlling the appropriate levels and cellular distribution of proteins needed for ribosome maturation is important and highly energy-dependent (Bohnsack and Bohnsack, 2019). Abnormal ribosome assembly caused by various pathophysiological conditions (such as changes in nutrient levels, stresses, genetic mutations) may result in various diseases (Wong et al., 2011; Pelava et al., 2016; Bohnsack and Bohnsack, 2019; Shen et al., 2019) and can be affected by many factors, including aging and genetic defects (Correll et al., 2019). Regulatory degradation of ribosomes by ribophagy (Oka and Yoneda, 2018) and the subsequent suppression of protein translation upon stresses (e.g., starvation) are also very important for cellular survival (von Walden, 2019; Yao et al., 2020).

Many initiation and elongation factors (e.g., eIF2, eIF4E, and eEF2) needed for protein translation from mRNA are known to be regulated by posttranslational modifications such as protein phosphorylation. Likewise, phosphorylation of the ribosomal protein S6 (rpS6), a component of the 40S ribosomal subunit, was reported many years ago (Gressner and Wool, 1974) and has been extensively investigated since then. It is an indicator of neuronal activity in many experimental models of neuronal plasticity and can be mediated by multiple kinases [including the p70 ribosomal protein S6 kinases (S6K1, S6K2), the p90 ribosomal protein S6 kinases (RSK), and protein kinase A (PKA); Knight et al., 2012; Meyuhas, 2015)]. Ample evidence suggests that the phosphorylation of rpS6 enhances protein translation. Additional extratranslational and extraribosomal roles of rpS6 and other ribosomal proteins have also been proposed (Biever et al., 2015).

Extensive studies have shown that hundreds of proteins are tightly coordinated to accomplish a successful protein translation process. These regulations are all energy-consuming processes (Roux and Topisirovic, 2018; Correll et al., 2019). For example, the functions of many enzymes (such as ATP-dependent RNA helicases, AAA-ATPases, GTPases, and kinases) involved in ribosome biogenesis and protein translation are highly energy-dependent (Kressler et al., 2010). Moreover, these processes can be fine-tuned during a wide variety of pathophysiological conditions (e.g., proliferation, differentiation, apoptosis, and degeneration). Poor translation efficiency consequently affects proteostasis, impairs cellular functions, and causes various diseases, including neurodegenerative diseases (Lehmkuhl and Zarnescu, 2018; Tahmasebi et al., 2018).

## Regulation of Protein Translation by AMPK

In response to energy stresses, AMPK is activated to restore energy homeostasis by the promotion of catabolic processes or/and the suppression of anabolic processes. It is a serine/threonine kinase that senses the cellular AMP/ATP ratio and positively controls its activation (Kemp et al., 1999). AMPK holoenzyme is composed of a catalytic α subunit and two regulatory subunits (β and γ), and can be regulated by the adenine nucleotide/energy sensing machinery or other upstream kinases (e.g., CamKKβ, TAK1) that are involved in metabolic responses under various pathophysiological conditions, including elevated levels of reactive oxygen species (ROS) that suppress mitochondrial ATP synthesis (Hardie et al., 2012; Ross et al., 2016; Gonzalez et al., 2020). Earlier studies showed that AMPK activity is high in the brain during development (Ramamurthy and Ronnett, 2006; Ronnett et al., 2009). This is of great interest because AMPK is activated following synaptic activation to maintain metabolic plasticity and energy level by enhancing glycolysis and mitochondrial respiration, suggesting a critical role of AMPK in controlling the neuroenergetic plasticity (Marinangeli et al., 2018). Dysregulation of AMPK either positively or negatively disrupts this synaptic plasticity in response to neuronal activation and may induce synaptic loss and impair cognitive functions (Marinangeli et al., 2018; Domise et al., 2019; Wang et al., 2019). Given that aberrant AMPK activation due to energy deficiency has been reported in degenerating neurons of several neurodegenerative diseases (including Alzheimer’s disease AD; Chen et al., 2009; Mairet-Coello et al., 2013; Ma et al., 2014; Chang et al., 2021), Huntington’s disease (HD; Ju et al., 2011, 2014), and amyotrophic lateral sclerosis (ALS; Lim et al., 2012; Liu et al., 2015a,b), a better understanding of the function and regulation of AMPK during neurodegeneration may provide potential targets for the development of new treatments for neurodegeneration diseases.

The role of AMPK in the control of protein translation has also attracted much attention lately. Given that AMPK activation inhibits protein translation by multiple pathways (Figure 1B) as detailed in the following sections, inferior protein translation in neurodegenerative diseases is a critical issue worthy of further investigation.

### Direct Regulation of Protein Translation by AMPK

Many studies have demonstrated that the protein translation process is controlled by energy homeostasis in a coordinated manner (Leibovitch and Topisirovic, 2018). Suppression of protein translation by AMPK at multiple steps has been reported. For example, the γ2-containing AMPK complex protected cells from ischemia-evoked injury by entering the nuclei to suppress the production of pre-rRNA and ribosome biosynthesis (Cao et al., 2017). In addition, AMPK directly phosphorylates eukaryotic elongation factor 2 kinase (eEF2K), which in turn phosphorylates eEF2 to suppress peptide translocation during protein translation and thus inhibits the rate of protein translation (Browne et al., 2004).

### Regulation of the Mechanistic Target of Rapamycin Complex 1 (mTORC1) by AMPK

The mammalian target of rapamycin (mTOR) signaling pathway is a key pathway that integrates multiple signals for cell growth and differentiation and controls protein translation (Laplante and Sabatini, 2009). mTOR is a serine-threonine kinase and a member of the PI3K kinase family. It interacts with four other proteins (including raptor) and forms mTOR complex 1 (mTORC1). During activation, mTORC1 activates S6K1 by phosphorylation and disinhibits eIF-4E by suppressing its repressor (4E-binding protein 1, 4E-BP; Lekmine et al., 2003), which subsequently facilitates protein translation (Figure 1B). Many of these regulatory pathways are linked to energy-dependent pathways. Specifically, mTORC1 plays a critical role in coordinating protein translation and ATP production (Leprivier et al., 2013). Activation of mTORC1 suppresses 4E-BPs and leads to selective translation of mitochondrial proteins and enhanced mitochondrial biogenesis, thus forming a link between protein translation and mitochondrial function (Morita et al., 2013).

Ample evidence suggests that AMPK indirectly suppresses the initiation of protein translation *via* the inhibition of mTORC1 signaling through the phosphorylation of mTOR, raptor, and a negative regulator of mTORC1 (i.e., TSC2; Bolster et al., 2002; Inoki et al., 2003; Gwinn et al., 2008). Conversely, under pathological conditions (e.g., nutrient stress), TORC1 directly phosphorylates the catalytic subunits of AMPK and inhibits AMPK activation (Ling et al., 2020). Such reciprocal feedback regulation between two major metabolic signaling pathways provides an emerging opportunity for the development of therapeutic strategies for various human diseases (Holczer et al., 2019; Gonzalez et al., 2020; Ling et al., 2020).

### Stress Granules and AMPK

In response to various stresses, eukaryotic cells form membraneless stress granules (SGs) containing a wide variety of translationally stalled mRNAs and mRNA-binding proteins (RBPs) in the cytoplasm to suppress protein translation (Buchan and Parker, 2009; Protter and Parker, 2016) and to control cellular metabolism and survival (Advani and Ivanov, 2020). These SGs appear to be heterogeneous and contain proteins of various functions, including those involved in translation initiation, mRNA degradation machinery, and other regulatory pathways for RNA metabolism. Many proteins enriched in SGs contain intrinsically disordered regions (IDRs), which not only facilitate diverse protein-protein and protein-RNA interactions but also control the assembly of SGs (Mittag and Parker, 2018). Accumulating evidence suggests that the dynamics of SGs may contribute to neurodegeneration (Advani and Ivanov, 2020).

Activation of AMPK is directly linked with SG formation. During elevated oxidative stress, AMPKα2 and the βγ subunits associate with G3BP1, a core component of SGs, and facilitate the formation of SGs. Recent studies suggest that, in response to stresses, G3BP1 coordinates SG assembly in an RNA-dependent, liquid-liquid phase separation (LLPS)-mediated manner (Yang W. et al., 2020). Suppression of AMPK using a specific inhibitor (i.e., compound C) or expression of a dominant-negative mutant of AMPKα2 demonstrated that AMPK activity is required for the formation of SGs (Mahboubi et al., 2015a,b; Kuo et al., 2020). Although AMPK activation enhances SG biogenesis, it limits the fusion of SGs and thus restrains the size of SGs (Mahboubi et al., 2016). Collectively, AMPK activation upon stress initiates the reprogramming of protein translation and RNA metabolism by facilitating SG assembly. It is interesting to note that G3BP1 also interacts with 40S ribosomal subunit proteins (e.g., rpS6) in SGs and may contribute to the global suppression of protein translation (Kedersha et al., 2016; Panas et al., 2016).

Among the many substrates of AMPK, importin-α1 plays a critical role in nucleocytoplasmic transport (Oka and Yoneda, 2018). A recent study reported that transient arsenite-mediated stress may trigger the formation of cytosolic liquid droplets of TDP-43, a pathological hallmark of ALS, and recruit importin-α1 and other components of nucleocytoplasmic transport to suppress the function of nucleocytoplasmic trafficking (Gasset-Rosa et al., 2019). Phosphorylation of importin-α1 at Ser^105^ by AMPK blunts its ability to transport a few nuclear RNA-binding proteins (including HuR and FUS) and to alter their cellular distributions (Wang et al., 2004; Liu et al., 2015b). HuR is an RNA-binding protein that binds with and stabilizes ARE-containing mRNA and is also a marker for SGs (Gallouzi et al., 2000). Importantly, importin-α1 has also been implicated in SG assembly during various stresses (e.g., elevated oxidative stress, sodium arsenite, and heat shock). It indirectly binds to poly(A)-containing RNA and localizes to SGs during stress for the preservation of cellular functions (such as cell survival and stress resistance; Fujimura et al., 2010; Mahboubi et al., 2013).

## Impaired Translational Control Resulting from Energy Dysfunction in Neurodegenerative Diseases

The mitochondrial defect that causes energy deficiency in neurons is a major pathological feature of most neurodegenerative diseases and has been extensively reviewed recently (Monzio Compagnoni et al., 2020; Yan et al., 2020; Goyal and Chaturvedi, 2021; Johnson et al., 2021; Lee et al., 2021; Madruga et al., 2021; Nakagawa and Yamada, 2021). We have previously reviewed the evidence linking metabolic impairment and mitochondrial defect in neurodegenerative diseases (Liu and Chern, 2015; Liu et al., 2017). The current review focuses on an emerging interest in the abnormal modulation of protein translation, a key element of proteostasis, by energy dysfunction in the context of two degenerative diseases (i.e., ALS and AD) as detailed in Table 1 and in the text below.

**Table 1:** Aberrant translational control in ALS and AD.

Disease	Disease-causing pathway(s)/ molecule(s)	Model	Functional impact	References
ALS	TDP-43	HEK293E cells, Sh-SY5Y cells	TDP-43 deficiency enhances global translation by alternative splicing of *SKAR* and enhancing S6K1 signaling.	Fiesel et al. (2012)
ALS	TDP-43	HEK293E, Sh-SY5Y cells	TDP-43 functions as a mRNA-specific translational enhancer for *Camta1*, *Mig12*, and *Dennd4a*, and contributes to neurodegeneration.	Neelagandan et al. (2019)
ALS	C9orf72 dipeptide repeat	Human iPSC-derived motor neurons, adult Drosophila neurons	Poly(GR) and poly(PR) interact with ribosomal proteins to induce a toxic translational arrest, which can be rescued by overexpression of eIF1A.	Moens et al. (2019)
ALS	C9orf72 dipeptide repeat	Drosophila, patient-derived fibroblasts, postmortem brain tissues	Patients harboring the G4C2 mutation have lower levels of eIF4H than control subjects. Downregulation of eIF4B and eIF4H reduced toxicity caused by poly(GR), suggesting that eIF4B and eIF4H are disease modifiers.	Goodman et al. (2019)
ALS	C9orf72 dipeptide repeat	Poly(GR) transgenic mice, HEK293T cells	Poly(GR) impairs protein translation by interacting with ribosomal proteins (e.g., rpS6) and eIF3*β*, and interfering with SGs dynamics.	Zhang et al. (2018)
ALS	C9orf72 dipeptide repeat	NSC34 cells, primary cerebral cortical neurons, HEK293 cells.	Poly(PR) inhibits the DEAD-box RNA helicases, and thus reduces the level of ribosomal RNAs and ribosome biogenesis, and triggers neuronal cell death.	Suzuki et al. (2018)
ALS	C9orf72 dipeptide repeat	Human iPSC-derived motor neurons	Poly(GR) interacts with mitochondrial ribosomal proteins and causes abnormal mitochondrial translation.	Lopez-Gonzalez et al. (2016)
ALS	C9orf72 dipeptide repeat	NSC34 cells, human brain tissues	Poly(PR) and poly(GR) jeopardize global protein translation by interacting with eIF3A, eEF1A, and RNA-binding proteins (such as hnRNPA1, TDP-43, and FUS), and forming insoluble complexes with mRNA.	Kanekura et al. (2016)
ALS	C9orf72 dipeptide repeat	HEK293 cells, SH-SY5Y cells	Poly(PR) and poly(GR) peptides enter the nucleolus to cause nucleolar stress, suppress the synthesis of ribosomal RNA, and contribute to neurodegeneration.	Tao et al. (2015)
ALS	C9orf72 GGGGCC (G4C2) repeats	NSC34 cells, SH-SY5Y cells, HeLa cells	RNA foci composed of C9orf72 GGGGCC (G4C2) repeat sequester translational regulators, impair nuclear mRNA export, and affect translation efficiency.	Rossi et al. (2015)
ALS	SOD1	Motor neurons of SOD1-G93A mice, patient-derived fibroblasts	SOD1-G93A alters protein translation by changing ribosome composition	Szelechowski et al. (2018)
ALS	SOD1	SOD1-G37R mice	Suppression of ribosomal genes and components of the translation machinery.	Sun et al. (2015)
ALS	FUS	N2A cells, patient-derived fibroblasts	ALS-related FUS mutants (e.g., R521G, P525R) impair protein translation by causing ribosomal stalling.	Kamelgarn et al. (2018)
AD	Tau	HEK cells, brain tissues of rTg(tauP301L)4510 mice and AD patients	Tau interacts with rpS6 and specifically suppresses protein translation in AD.	Koren et al. (2019)
AD	n/a	Brain tissues of AD patients	AD patients have abnormal regulation of ribosomal proteins (e.g., RPS5, RPS6, RPS7, RPS13), initiation factors (eIF2α, eIF5), and elongation factors (eEF2).	Hernandez-Ortega et al. (2016)
AD	n/a	Brain tissues of AD patients	AD patients have lower levels of ribosomal RNA and tRNA, and inferior capacity for protein synthesis.	Ding et al. (2005)
AD	n/a	Brain tissues of AD patients	AD patients have abnormal phosphorylation levels of translational regulators (e.g., mTOR, 4E-BP1, eEF2K), suggesting aberrant protein synthesis.	Li et al. (2005)
AD	APP	Brain tissues of AD patients and Tg19959 AD mice	The dysregulation of eEF2K/eEF2 signaling in AD brains is a drug target of AD.	Beckelman et al. (2019)
AD	A*β*42 oligomer	Brain tissues of AD patients and APP/PS1 mice	AD patients have higher eEF2K activity, suggesting that eEF2K is a drug target of AD.	Jan et al. (2017)
AD	A*β*42 oligomer	Brain tissues of AD patients and APP/PS1 mice	AD patients have abnormal levels of ribosomal RNAs (rRNA18S, rRNA28S), ribosomal proteins (rpS5, rpS6, rpS10, rpS13), initiation factors (eIF3eta), and elongation factors (eEF1A, eEF2), suggesting aberrant protein synthesis.	Garcia-Esparcia et al. (2017)
AD	Tau	Brain tissues of AD patients, mouse primary neurons	Tau associates with ribosomes and impairs protein translation (e.g., of PSD-95).	Meier et al. (2016)
AD	A*β*42 oligomer, Tau	Brain tissue of AD patients and 3 × Tg AD mice	The level of S6K1 is higher in AD brains and is associated with the accumulation of A*β* and Tau. S6K1 is a drug target of AD.	Caccamo et al. (2015)
AD	A*β*42 oligomer	Brain tissues of AD patients and APP/PS1 mice	A*β* oligomers evoke AMPK activation, which causes detrimental effects *via* the regulation of translational regulators (eEF2 and eEF2K) and contribute to AD-related synaptic pathogenesis.	Ma et al. (2014)
AD	A*β*42 oligomer	Brain tissues of AD patients, rat primary neurons	A*β*42 activates AMPK, which subsequently inhibits the mTOR pathway, and inhibits protein translation.	Yoon et al. (2012)
AD	Tau	Brain tissues of AD patients, rat primary neurons, SH-SY5Y cells	Upregulation of the p70S6K in the AD brain results in an increased expression and phosphorylation of Tau and contributes to PHF-Tau accumulation and neurodegeneration.	An et al. (2003)

*AD, Alzheimer’s disease; ALS, amyotrophic lateral sclerosis; AMPK, AMP-activated protein kinase; APP, amyloid precursor proteins; A*β*42, Amyloid *β*42; DPR, dipeptide repeat; FUS, fused in sarcoma; n/a, not applicable; Poly(GA), glycine-alanine repeat protein; NFTs, neurofibrillary tangles; PHF, paired-helical-filament; Poly(GR), glycine-arginine repeat protein; Poly(PR), proline-arginine repeat protein; rpS6, ribosomal protein S6; S6K1, ribosomal S6 kinase 1; SGs, stress granules; SKAR, the ribosomal S6 kinase 1 (S6K1) Aly/REF-like target; TDP-43, TAR DNA-binding protein 43*.

### Amyotrophic Lateral Sclerosis (ALS)

ALS is a progressive motor neuron disease characterized by the degeneration of motor neurons. The loss of motor neurons leads to muscle weakness, disability, and shortened life span. The common hallmark of familiar and sporadic ALS is the mislocalization and accumulation of cytoplasmic inclusions containing the TAR DNA-binding protein 43 (TDP-43) in motor neurons. TDP-43 has diverse functions, including RNA splicing, mRNA transport, protein translation, and storage of specific mRNAs in SGs (Buratti et al., 2001; Prasad et al., 2019). For example, TDP-43 is known to facilitate miRNA biogenesis that is critical for neuronal outgrowth by interacting with Drosha and Dicer complexes (Kawahara and Mieda-Sato, 2012). The mislocalization of TDP-43 in motor neurons is increased that is associated with energy dysfunction and AMPK activation (Liu et al., 2015a). The cellular redistribution of TDP-43 has been implicated in the impairment of translation machinery. For example, in a Drosophila model of ALS, TDP-43 binds to futsch mRNAs and switches actively translating polysomes to nontranslating ribonuclear protein particles (Coyne et al., 2014). The mislocalized mutant TDP-43 in the cytoplasm also disrupts translational processing of hsc70-4 by recruiting mRNA from translating ribosomes (Coyne et al., 2017). Such translational inhibition also caused the impairment of synaptic vesicle endocytosis (Estes et al., 2011). In mouse hippocampal neurons, TDP-43 mediates neuronal spinogenesis *via* translational repression of Rac1 (Majumder et al., 2012). Interestingly, TDP-43 can regulate global translation by controlling the expression of S6 kinase 1 Aly/REF-like target (SKAR) in human embryonic kidney cells. SKAR is a downstream molecule of the mTOR/S6K1 pathway, which plays a critical role in controlling cellular translation (Fiesel et al., 2012). This is of great interest because AMPK can regulate translation by controlling the activity of mTOR (Garza-Lombo et al., 2018). Most importantly, AMPK is abnormally activated in the motor neurons of patients and two mouse models with ALS, which causes the mislocalization of TDP-43 (Lim et al., 2012; Liu et al., 2015a). It is very likely that, in addition to the AMPK-mTOR axis, AMPK might also mediate cellular translation machinery by altering TDP43-mediated translation in ALS.

In addition to TDP-43, C9orf72-encoded hexanucleotide repeat expansions have been demonstrated to be a critical molecule in ALS pathology (DeJesus-Hernandez et al., 2011; Renton et al., 2011). The toxicity mediated by dipeptide repeats (DPRs) encoded by C9orf72 *via* repeat-associated non-AUG (RAN) translation has attracted much attention. The RAN translation of C9-G4C2 repeats results in poly(PA), poly(PR), poly(GA), poly(GP), and poly(GR) dipeptides. Translational initiation factors (such as eIF4B and eIF4H) are important for the RAN translation of poly-GR production (Goodman et al., 2019). These C9-DPRs have been directly associated with the impairment of translation in ALS. For example, Zhang et al. (2018) have shown that C9-poly(GR) DPRs impair protein translation by interacting with ribosomal protein (rpS6) and translational initiation factor eIF3*β* in poly(GR) transgenic mice and ALS patients. In a Drosophila ALS model and human iPSC-derived motor neurons, C9 arginine-rich dipeptide repeats disrupted ribosomal biogenesis by interacting with ribosomal proteins such as rpS23, rpS30, rpL5, and rpL36 (Moens et al., 2019). Furthermore, poly(GR) and poly(PR) also caused a toxic translational arrest that can be restored by exogenous expression of eIF1A (Moens et al., 2019). In a motor neuron cell line, Kanekura et al. (2016) demonstrated that C9-arginine-rich DPRs impaired protein translation by sequestering translation factors (eIF3A, eEF1A; Simov et al., 2016). Moreover, in NSC34 cells, Suzuki et al. (2018) also indicated that C9-poly(PR) disrupted translational machinery by reducing ribosomal RNA expression levels. In addition to altering the levels of ribosomal RNAs, this study also shows that C9-poly(PR) impaired ribosome biogenesis and triggered neuronal toxicity by interacting with DEAD-box RNA helicases (Suzuki et al., 2018). Of note, the nucleolus is an important cellular organelle of ribosome assembly that produces ribosomal RNA (rRNA) and forms pre-ribosome subunits. Abnormal nucleolar stress may impair translation machinery by reducing rRNA biogenesis, ribonucleoprotein binding, and ribosome assembly (Yang et al., 2018). Expression of C9-DPRs leads to nucleolar stress by being localized to the nucleolus and suppressing ribosomal RNA biogenesis (Tao et al., 2015), suggesting that C9-DPRs may also impair translational machinery by inducing nucleolar stress.

It is of great interest to note that C9-mediated DPRs have been implicated in the disruption of cellular energy homeostasis by damaging mitochondrial functions (Onesto et al., 2016; Choi et al., 2019). For example, C9-mediated poly(GR) selectively binds to ATP5A1 (a mitochondrial complex V component) to reduce its expression and causes toxicity in neurons of patients and mice with C9-poly(GR) (Choi et al., 2019). In addition, C9orf72 is a mitochondrial protein. The loss of C9orf72 impairs its function to maintain the integrity of the respiratory chain (Wang et al., 2021). Recent studies also showed that motor neurons derived from iPSCs of *C9orf72*-ALS patients have inferior mitochondrial bioenergetic functions, poor calcium buffering ability, and shorter axons (Dafinca et al., 2020; Mehta et al., 2021). Collectively, mitochondrial defects and the resultant defective energy metabolism is believed to be a major driver that plays a critical role in ALS pathogenesis and was extensively discussed in recent reviews (Calió et al., 2020; Dafinca et al., 2021; Jhanji et al., 2021; Madruga et al., 2021; Nakagawa and Yamada, 2021). The possible role of energy deficiency and AMPK activation in the impairment of protein translation in ALS warrants further research.

The formation of SGs has attracted much attention in ALS. SGs are transient organelles that regulate cellular RNA homeostasis and translational pathways (Buchan and Parker, 2009). SGs have been indicated to cause the inactivation of protein translation by sequestering RBPs, mRNA, and ribosomal proteins under stress conditions (Buchan and Parker, 2009; Protter and Parker, 2016). Many studies have suggested a critical relationship between SGs and ALS pathology. First, several RBPs associated with ALS are localized in SGs. For example, FUS has been shown to be localized to SGs under oxidative stress or heat shock conditions (Bosco et al., 2010). Interestingly, TDP-43 can also be sequestered into SGs during several forms of stress (i.e., oxidative stress, ER stress; McDonald et al., 2011). In the spinal cords of ALS patients, TDP-43 aggregates were found to be colocalized with SG markers (such as TIA-1 and eIF3; Liu-Yesucevitz et al., 2010). Fang et al. (2019) reported that transient SG formation prevents persistent TDP-43 accumulation in iPS-MNs from ALS patients by recruiting TDP-43 into SGs. In addition, a recent study showed that cytoplasmic TDP-43 forms liquid droplets to inhibit nucleocytoplasmic transport, which is independent of SGs (Gasset-Rosa et al., 2019). Moreover, ALS-related ribonucleoproteins, such as hnRNP A1 and hnRNP A2B1, are components of SGs under stress stimulation (Guil et al., 2006; Kim et al., 2013). Notably, TDP-43 also has a physiological function in SG assembly (McDonald et al., 2011). Downregulation of TDP-43 can delay SG formation, while the loss of this function is associated with ALS pathology (Aulas et al., 2012). In addition to RBPs, another disease-causing protein (mutant SOD1) is also localized in SGs. mSOD1 interacts with G3BP1 and then interferes with the assembly of SGs (Gal et al., 2016). Moreover, C9-DPRs can interact with an SG marker (G3BP1) to impair the assembly and function of SGs (Lee et al., 2016). Importantly, the poly-PR and poly-GR of C9-DPRs also enhance SG formation and delay cellular protein translation (Wen et al., 2014; Kanekura et al., 2016). Collectively, SG formation is an important feature of ALS pathology, which is associated with the dysfunction of RBPs and the impairment of cellular translation machinery.

### Alzheimer’s Disease (AD)

Protein translation is required for maintaining long-lasting synaptic plasticity and memory (Klann and Dever, 2004). Accumulating evidence suggests that poor protein translation is associated with dementia. Abnormal activation of AMPK was reported in the hippocampus of aged mice (Yang et al., 2019). Moreover, reduced expression of many ribosome-related proteins (ribosomal protein large and small subunits) was differentially regulated in the aged hippocampus of primates (Meng et al., 2020), suggesting tight control of protein translation, particularly local translation in synapses, during the aging process.

AD is the most common neurodegenerative disease, contributing to 60–70% of dementia in the world (Organization, 2020). Clinical presentations include (but are not limited to) the loss of recent memory, mood swings, and disorientation. Major pathological hallmarks of AD are amyloid plaques, hyperphosphorylation-Tau containing neurofibrillary tangles, vascular impairment, and chronic inflammation. Dysfunction of protein synthesis has been reported in brains associated with AD. For example, lower ribosomal functions and reduced ribosomal levels were shown in the cortical areas of MCI and AD patients, suggesting that impaired protein synthesis is an early AD pathology and may contribute to the development of AD (Ding et al., 2005). Altered levels of ribosomal proteins (e.g., rpS5 and rpS6) and initiation factors (e.g., eEF2) were also observed in the brains of mice and in patients with AD (Hernandez-Ortega et al., 2016; Garcia-Esparcia et al., 2017). A combination of translating ribosome affinity purification (TRAP) and RNA sequencing analyses also revealed that ribosomal processing pathways are downregulated in a mouse model of AD (McKeever et al., 2017). Collectively, protein synthesis gradually becomes abnormal during AD progression.

Abnormal regulation of AMPK and dysregulated mTOR pathways were both found in brains affected by AD. Amyloid β_42_ impairs translation by AMPK activation, thereby blocking the mTOR pathway (Yoon et al., 2012). Furthermore, the mTOR downstream target S6K1 increases the translation of *tau* mRNA by phosphorylating rpS6 in SH-SY5Y cells (Pei et al., 2006). Conversely, the reduction in S6K1 levels in an AD mouse model results in the inhibition of Tau translation. Such a reducing effect of S6K1 can improve spatial memory and synaptic plasticity (Caccamo et al., 2015). Moreover, eEF2 and its kinase eEF2K have been indicated to be the key downstream molecules of AMPK in AD pathology. eEF2K is known to phosphorylate and suppress eEF2 so that the rate of protein synthesis is reduced (Proud, 2015). Genetic inhibition of eEF2K enhances the translation machinery and improves compromised long-term potentiation (LTP) in AD mice (Beckelman et al., 2019). Importantly, treatment with an AMPK inhibitor (compound C) improves the decreased synaptic plasticity in AD mice (Ma et al., 2014). Because enhanced AMPK activity is associated with poor neuronal plasticity, suppression of AMPK activity was proposed to be a therapeutic strategy (Wang et al., 2019).

AMPK also directly phosphorylates Tau (Domise et al., 2016). While hyperphosphorylated Tau drives the formation of neurofibrillary tangles as a major hallmark of AD, Tau was found to impair protein translation by disrupting ribosome biogenesis by interacting with rpS6 in the brains of AD patients and mice with tauopathy (Koren et al., 2019). Moreover, phosphorylated Tau protein (pTau) interacts with the splicing factor proline and glutamine rich (SFPQ) and is localized in SGs in the brains affected by rapidly progressive AD (Younas et al., 2020). In addition, pathogenic Tau has been shown to associate with several ribosomal proteins (e.g., rpS5) and to selectively affect protein translation (Meier et al., 2016; Koren et al., 2019). For example, the synthesis of PSD95 has been reduced by Tau (Meier et al., 2016).

SGs play critical roles in AD pathology and are involved in RNA metabolism and translational control. The formation of SGs controls the translation process by sequestering cellular RNA and RBPs (Ivanov et al., 2019). The accumulation of Tau proteins accelerates SG formation by interacting with TIA-1 (Vanderweyde et al., 2016). The expression of human Tau *in vitro* is known to cause the downregulation of an RNA-binding protein (splicing factor proline and glutamine rich, SFPG). Under oxidative stress, SFPQ is recruited to SGs and colocalizes with Tau. Such dysfunction of SFPQ is associated with rapidly progressive AD patients (Younas et al., 2020). In line with the importance of SGs in AD, chronic stress and high glucocorticoid (GC) levels disrupt protein homeostasis, enhance the accumulation of RNA binding proteins residing in SGs, and facilitate Tau aggregation in a transgenic Tau mouse model of AD (Silva et al., 2019). Taken together, ample evidence demonstrates that abnormal translation control, AMPK activation, and SGs are involved in AD pathology. Further studies are required to explore the possible involvement of abnormal interplay between energy homeostasis and protein translation in AD pathogenesis.

## Conclusion Remarks

The protein translation process is complex and highly energy- dependent. In the past three decades, the translation machinery has been extensively studied at the molecular level. Dysregulated protein translation in neurodegenerative diseases may occur by a wide variety of complex mechanisms that lead to inferior ribosome biogenesis and poor protein synthesis efficiency. Recently, interventions that enhance protein translation efficiency have been linked with improved neuronal plasticity and functions (e.g., cognitive activities) in many experimental models of neurodegenerative diseases. AMPK is considered a master regulator of energy homeostasis and its activation has been reported in various stages of neurodegenerative diseases. To date, many direct or indirect activators of AMPK have been developed, and some are currently being evaluated for their contribution to human diseases and disorders (mostly metabolic diseases, inflammation-associated diseases, and cancers; Steinberg and Carling, 2019; de Souza Almeida Matos et al., 2019). For AD, the effects of AMPK activators remain controversial. As reviewed recently by Assefa et al. (2020), most AMPK activators evaluated in AD are indirect activators (such as resveratrol and metformin). In addition to AMPK, these compounds also have other targets important for neuronal functions and survival. Furthermore, different AMPK subtypes (e.g., α1 vs. α2) were recently shown to play seemingly opposite roles in the control of cognitive functions (Yang P. et al., 2020; Zimmermann et al., 2020). Such complex factors may contribute to the observations that both positive and negative effects of AMPK activators on AD pathogenesis (including amyloid metabolism and tauopathy) have been reported. These exciting findings suggest that in-depth investigation into the roles of AMPK in the context of neurodegenerative diseases is required. Moreover, the upstream regulators of AMPK may also serve as excellent drug targets. For example, a new antioxidant drug (Edaravone) was recently approved for ALS (Cho and Shukla, 2020; Shakkour et al., 2021). Many natural antioxidants (such as curcumin, ginkgo biloba) have also been tested in preclinical studies and clinical trials of AD (Arslan et al., 2020). Future detailed investigations in the context of the AMPK-mediated control of protein translation efficiency will better illuminate the specific steps/pathways of protein synthesis that are sensitive to energy deficiency as well as the spectrum of strategies that can be applied to the development of novel therapies for neurodegenerative diseases. To this end, the identification of mislocalized proteins caused by the AMPK-mediated impairment of nuclear–cytoplasmic shuttling, the molecular understanding of stalled translation complexes by disease-causing proteins in SGs, and the discovery of genetic modulations/pharmacological tools to preserve sufficient protein synthesis in an energy crisis will provide critical features of the association between energy dysfunction and neurodegeneration, and may pave the way to identify new drugs for neurodegenerative disease.

## Author Contributions

Y-JL and YC both wrote parts of the mini review. All authors contributed to the article and approved the submitted version.

## Conflict of Interest

The authors declare that the research was conducted in the absence of any commercial or financial relationships that could be construed as a potential conflict of interest.

## Publisher’s Note

All claims expressed in this article are solely those of the authors and do not necessarily represent those of their affiliated organizations, or those of the publisher, the editors and the reviewers. Any product that may be evaluated in this article, or claim that may be made by its manufacturer, is not guaranteed or endorsed by the publisher.
